# Bovine Lactoferrin Inhibits Pseudorabies Virus Attachment Through Blockade of Glycoprotein C‐Heparan Sulfate Interaction

**DOI:** 10.1155/tbed/1942686

**Published:** 2026-07-08

**Authors:** Yiping Wang, Binbin Zhu, Fei Zhao, Zhiyuan Zheng, Senhong Zhao, Yi Zheng, Xiaobo Huang, Qin Zhao, Senyan Du, Yiping Wen, Rui Wu, Tongqing An, Sanjie Cao

**Affiliations:** ^1^ Research Center for Swine Diseases, College of Veterinary Medicine, Sichuan Agricultural University, Chengdu, China, sicau.edu.cn; ^2^ Agricultural Animal Diseases and Veterinary Public Health Key Laboratory of Sichuan Province, Sichuan Agricultural University, Chengdu, China, sicau.edu.cn; ^3^ Engineering Research Center of Southwest Animal Disease Prevention and Control Technology of Ministry of Education, Sichuan Agricultural University, Chengdu, China, sicau.edu.cn; ^4^ Key Laboratory of Agricultural Bioinformatics of Ministry of Education, Sichuan Agricultural University, Chengdu, China, sicau.edu.cn; ^5^ State Key Laboratory of Animal Disease Control and Prevention, Harbin Veterinary Research Institute, Chinese Academy of Agricultural Sciences, Harbin, China, caas.cn

**Keywords:** bovine lactoferrin, glycoprotein C, heparan sulfate, pseudorabies virus, virus attachment

## Abstract

Pseudorabies virus (PRV) is an important swine pathogen that causes significant economic losses in the pig industry. In addition to its swine host, PRV also infects a wide variety of animals as well as humans, posing a threat to veterinary public health. Thus, the development of potent antiviral drugs against PRV is required. Bovine lactoferrin (BLF) is widely known as an iron‐binding glycoprotein in the transferrin family, which gains great attention for biomedical applications due to its beneficial physiological functions to human health as an antioxidant, antimicrobial, antiviral, anticancer, and immunomodulatory agent. In the work described here, we identified BLF as a novel and potent antiviral agent against PRV. BLF exhibited strong antiviral activity against PRV infection in multiple pig, human, and mouse permissive cells. Systematic analysis of the effect of BLF on PRV inhibition revealed that BLF blocks PRV attachment to target cells. Through surface plasmon resonance (SPR) analysis, BLF was demonstrated to directly interact with heparan sulfate (HS) proteoglycan (HSPG), a primary attachment receptor for PRV. Addition of exogenous HS completely abolished BLF inhibition of PRV attachment, demonstrating that BLF represses PRV attachment by interacting with cell‐surface HS. However, BLF failed to inhibit the attachment of a PRV mutant virus with the deletion of glycoprotein C (gC), the primary viral envelop component for HS binding, suggesting that PRV gC is required for BLF inhibition of PRV infection. Cumulatively, these findings demonstrated that BLF inhibits PRV attachment by binding cell‐surface HS to block the interaction between viral gC and HS.

## 1. Introduction

The pseudorabies virus (PRV), also known as the suid herpesvirus 1 or Aujeszky’s disease virus, is an economically important swine pathogen that causes fatal encephalitis in newborn piglets and severe reproductive failure in pregnant sows, leading to enormous economic losses in the pig industry worldwide [1, 2]. PRV has a wide host range, with the capability to infect a large variety of animals, including cattle, horses, goats, sheep, chickens, rabbits, deer, raccoons, possums, skunks, ferrets, wolves, dogs, cats, guinea pigs, and rodents, in addition to its natural swine host [1, 2]. Aside from pigs, infection of other animals by PRV often causes severe neurological symptoms and leads to death [1, 3]. Historically, PRV infection in humans has been controversial because the researchers failed to isolate the virus from PRV‐infected laboratory personnel and pig farm workers, with only PRV antibodies and DNA being detectable [4–12]. Until recently, the first human PRV strain hSD‐1/2019, was isolated from the cerebrospinal fluid of PRV‐infected patients, demonstrating the capability of PRV to infect humans [13]. PRV infection of humans causes mild to severe clinical symptoms, such as sore throat, fatigue, fever, sweating, tinnitus, endophthalmitis, and encephalitis [4–13]. Due to the ability to infect various animals and humans, PRV holds a high risk of interspecies transmission [14, 15]. Consequently, PRV is not only a serious veterinary problem but also a potential public health concern.

PRV belongs to the genus *Varicellovirus* in the family Herpesviridae [16, 17]. It has a double‐stranded DNA genome of about 143 kb long, encoding over 70 viral genes [1]. According to the temporal expression, these viral genes can be divided into three distinct kinetic groups: immediate early genes (e.g., *IE180*), early genes (e.g., *UL42*) and late genes (e.g., *gB*). To initiate infection successfully, PRV must attach to target cells first by binding its encoded glycoprotein C (gC) to heparan sulfate (HS) proteoglycan (HSPG) on the cell surface [18–22]. PRV gC is the primary viral envelope component that binds cell‐surface HS [18–22]. Following initial attachment, PRV engages a non‐HS receptor, such as nectin 1, through gD, to be firmly tethered to the cell surface [23, 24]. The binding of PRV virions to host cells signals to the gH/gL heterodimer to activate the fusion property of the gB glycoprotein, which mediates the fusion between the virion envelope and the plasma membrane and facilitates viral entry [25–28]. It is noteworthy that while the cellular entry of the PRV wild‐type virus is gC‐dependent, the PRV gC‐deficient (PRVΔgC) virus can also enter target cells, suggesting that the entry of the PRVΔgC virus does not rely on cell‐surface HS [18, 19]. The gD glycoprotein on the gC‐deficient virions is thought to primarily bind cell‐surface receptors and mediate the subsequent virus entry process [18, 19]. Although the PRVΔgC virus can enter target cells effectively, it replicates at a significantly lower level as compared to the wild‐type virus [18, 19].

Lactoferrin (LF) is a naturally occurring iron‐binding glycoprotein in the transferrin protein family and is widely present in various physiological fluids [29]. Bovine LF (BLF) is primarily found in the secretions of mammary glands, including colostrum, transitional milk, and mature milk [29]. BLF is widely recognized for its broad‐spectrum biological activities, such as iron delivery, antioxidant, anti‐inflammatory, antitumor, antimicrobial, antiviral, immunomodulatory, and prebiotic functions [29]. These outstanding properties make it an attractive and promising candidate molecule for application in the fields of biomedicine and biotechnology. In support of this, BLF has been produced on a large scale and widely applied for infant formulas, dietary supplements, and functional food formulations [29, 30]. Not surprisingly, BLF has also been extensively investigated as a potential antiviral agent candidate against a variety of viruses, including herpes simplex virus type 1 (HSV1) and type 2 (HSV2), severe acute respiratory syndrome coronavirus (SARS‐CoV), SARS‐CoV‐2, human immunodeficiency virus (HIV), as well as hepatitis B virus (HBV) [31–33]. The antiviral mechanism of BLF is independent of its iron‐binding activity and is achieved primarily by blocking viral attachment through direct binding to viral particles or target cells [34–40]. However, to date, whether BLF exhibits antiviral activity against PRV infection remains completely unknown.

To date, no drugs are approved for use in animals or humans against PRV infection. To prepare for possible future PRV prevalence in humans, the development of effective drugs against PRV infection is required. In the work presented here, the antiviral efficacy of BLF against PRV infection was systematically investigated. We observed that BLF exhibited a strong inhibitory effect on PRV replication in multiple permissive cell lines derived from pigs, humans, and mice. Addition of BLF before and during but not after viral attachment significantly suppressed PRV replication, demonstrating that BLF inhibits PRV replication by blocking viral attachment. Further investigation revealed that BLF directly interacted with HSPG, and the addition of exogenous HS reversed the inhibitory effect of BLF on PRV attachment by competitively binding BLF. Notably, BLF did not affect the attachment of the PRVΔgC virus, which enters target cells in a HS‐independent manner. These results demonstrated that BLF blocks PRV attachment by interacting with cell‐surface HS to interfere with its binding to PRV gC. Cumulatively, these findings demonstrated that BLF is a novel antiviral agent against PRV infection that acts by interrupting the interaction between PRV gC and cell‐surface HS.

## 2. Materials and Methods

### 2.1. Cells and Viruses

PK15, Vero, Neuro‐2a, HeLa, and HuH7 cells were grown in Dulbecco’s modified Eagle medium (DMEM; Seven Biotech, SC102‐02) containing 10% fetal bovine serum (FBS; EXCell, FSP500) and 1x penicillin‐streptomycin solution (Beyotime, C0222) at 37°C and 5% CO_2_. The PRV wild‐type virus WLY06 strain, the PRVΔgC virus generated by replacing the PRV gC gene with green fluorescent protein (GFP) [41], and the PRV‐GFP marker virus generated by replacing the PRV TK gene with GFP were stored in our laboratory.

### 2.2. Cell Viability Assay

PK15, Vero, Neuro‐2a, HeLa, and HuH7 cells seeded in 96‐well plates were mock‐treated or treated with BLF (MedChemExpress, HY‐P3161, 0.0625, 0.125, 0.25, 0.5, and 1 mg/mL for PK15 cells; based on the toxicity test results of PK15 cells, 1 mg/mL of BLF without cytotoxicity was selected as the test concentration for other cell lines, including Vero, HeLa, HuH7, and Neuro‐2a cells) at 37°C for 24 h. Then, cell viability was determined using the CCK‐8 assay (CCK‐8, a WST‐8 based Cell Counting Kit‐8; Biosharp, BS350B).

### 2.3. Virus Infection

PK15, Vero, Neuro‐2a, HeLa, and HuH7 cells seeded in 12‐well plates (3 × 10^5^ cells/well) were mock‐treated or treated with BLF at the designated concentrations for 2 h. The cells were infected with PRV at a multiplicity of infection (MOI) of 0.01, 0.1, or 1 for 1 h with BLF and then incubated with BLF for the indicated time points. Subsequently, the viral titers in the cells and supernatants were determined by the plaque assay, the viral gene transcription and DNA synthesis in the cells were analyzed by reverse transcription‐quantitative PCR (RT‐qPCR) and qPCR, respectively, and the protein expression of viral genes in the cells was measured by western blots.

### 2.4. Plaque Assay

PK15 cells were seeded in 12‐well plates at 3 × 10^5^ cells per well. One day later, the viral samples harvested at the indicated time points were diluted, and the number of plaques was determined exactly as previously described [41].

### 2.5. RT‐qPCR and qPCR

RNA was extracted from PRV‐infected cells using the UE Multisource Total RNA Minprep Kit (US Everbright, UE‐MN‐MS‐RNA‐250), following the manufacturer’s instructions. The EasyScript All‐in‐One First‐Strand cDNA Synthesis SuperMix for qPCR (One‐Step gDNA Removal) (TransGen Biotech, AE341‐02) was used to synthesize cDNA with 1 μg of total RNA. The qPCRs were performed using the PerfectStart Green qPCR SuperMix (TransGen Biotech, AQ601‐02‐V2) with the primers specific for the PRV *gB* gene (forward: 5′—GACAACGAGCTCCTCATCTC—3′; reverse: 5′—ACGTAGCTGTAGTCCTCGTA—3′) and pig glyceraldehyde‐3‐phosphate dehydrogenase gene (GAPDH, forward: 5′—ACCTCCACTACATGGTCTAC—3′; reverse: 5′—GATGGCCTTTCCATTGATGA—3′). The expression of the PRV *gB* gene relative to GAPDH was detected using the comparative *C*_*T*_ method with GAPDH as an internal control [42].

DNA was extracted from PRV‐infected cells using the TIANamp Genomic DNA kit (TIANGEN BIOTECH, DP304‐03), following the manufacturer’s instructions. PRV genomic DNA was quantified by qPCR using the primers specific for the PRV *gB* gene (forward: 5′—GACAACGAGCTCCTCATCTC—3′; reverse: 5′—ACGTAGCTGTAGTCCTCGTA—3′), as described above. As an internal control, pig GAPDH DNA was determined with the following primers (forward: 5′—GACCTCAGCTCTTAGCAAAC—3′; reverse: 5′—CATCAGAGAACCATCCAGAAA—3′). The results were expressed as the relative PRV DNA levels normalized to GAPDH DNA.

### 2.6. Western Blots

The western blot assays were carried out exactly as previously described [41]. The primary antibodies used were anti‐PRV IE180 (1:50), anti‐PRV UL42 (1:5000, clone 7C11) [43], anti‐PRV gB (1:5000, clone 1E7), anti‐PRV gC (1:5000; DAYAO SHENGWU, Ab0104), and anti‐β‐actin (1:50,000; ABclonal, AC026).

### 2.7. Virus Attachment and Internalization Assay

The PRV attachment and internalization assay was performed exactly as previously described [41]. Briefly, the cells were treated with 1 mg/mL BLF at 37°C for 2 h, infected with PRV at MOI 1 at 4°C for 1 h, and then the virus attached to cells was quantified by qPCR. In the internalization assay, the cells were first infected with PRV at 4°C for 1 h, treated with 1 mg/mL BLF at 37°C for 2 h, and then the virus internalized into cells was measured by qPCR.

### 2.8. Surface Plasmon Resonance (SPR)

The SPR experiments were performed using a Biacore 8K machine with CM5 sensor chips (Cytiva, Marlborough, MA, USA) at MedChemExpress LLC (Shanghai). BLF was immobilized on the chips via amine coupling. Recombinant HSPG protein (Beijing Bioss, bs‐42231P, 100, 50, 25, 12.5, 6.25, and 3.125 nM) was administered into the channel with a flow rate of 30 μL/min. After each cycle, the sensor surface was regenerated using 10 mM glycine hydrochloride (pH 2.0). The binding kinetics and parameters for BLF and HSPG interactions were analyzed using Biacore Insight software (Cytiva) by fitting the data to a 1:1 Langmuir binding model.

### 2.9. Statistical Analyses

All data were analyzed with GraphPad Prism 9 software (GraphPad, San Diego, CA). Statistical significance was analyzed using unpaired Student *t*‐tests (two‐tailed), with *p* < 0.05 considered to be statistically significant.  ^∗^ *p* < 0.05;  ^∗∗^ *p* < 0.01;  ^∗∗∗^ *p* < 0.001; and  ^∗∗∗∗^ *p* < 0.0001.

## 3. Results

### 3.1. Identification of BLF as a New Antiviral Drug Against PRV Infection

**Figure 1:**
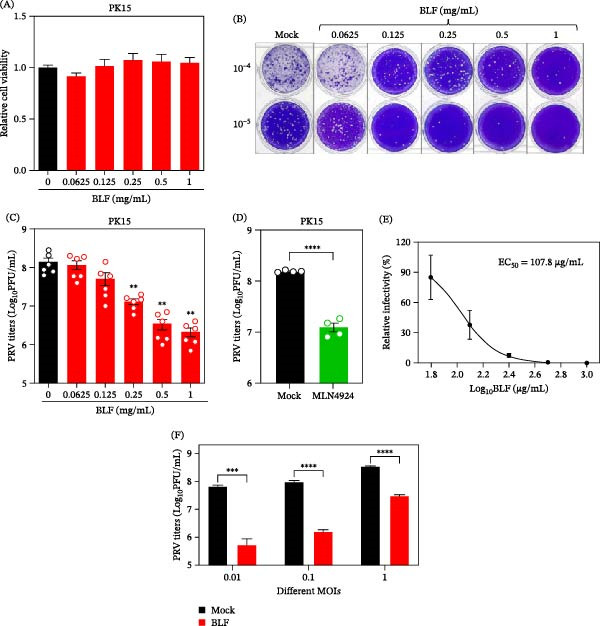
BLF inhibits PRV replication in a dose‐dependent manner. (A) PK15 cells were treated with BLF at 0, 0.0625, 0.125, 0.25, 0.5, or 1 mg/mL for 24 h, then the cell viability was measured by the CCK‐8 assay. (B, C) PK15 cells were mock‐treated, or treated with BLF at 0, 0.0625, 0.125, 0.25, 0.5, or 1 mg/mL for 2 h, infected with PRV at MOI 0.1 in the presence of BLF for 1 h, then incubated in the continued presence of BLF for 24 h. The viral titers in the cells and supernatants were determined by plaque assay. The representative plaque images from 10^−4^ and 10^−5^ virus dilutions are shown in (B), and the PRV titers were summarized in (C). (D) PK15 cells were infected with PRV at MOI 0.1, then mock‐treated or treated with MLN4924 at 10 µM for 24 h. The viral titers in the cells and supernatants were measured by plaque assay. (E) PRV infectivity in the presence of different concentrations of BLF relative to mock treatment was determined by plaque assay. The EC_50_ curve fitting was obtained using log_10_ (BLF concentrations) versus relative infectivity with variable slopes in GraphPad Prism 9 software. (F) PK15 cells were mock‐treated or treated with 1 mg/mL BLF for 2 h, infected with PRV at MOI 0.01, 0.1, or 1 in the presence of BLF for 1 h, and then incubated in the continued presence of BLF for 24 h. The viral titers in the cells and supernatants were detected by plaque assay. Values represent the means ± the standard errors of the mean (SEM) of two independent experiments. Significance was determined by a two‐tailed, unpaired *t*‐test ( ^∗∗^ *p* < 0.01,  ^∗∗∗^ *p* < 0.001,  ^∗∗∗∗^ *p* < 0.0001).

To determine whether BLF displayed an antiviral effect on PRV infection, we tested whether BLF repressed PRV replication in porcine kidney PK15 cells. To do this, we first measured the viability of PK15 cells treated with BLF at different concentrations (0.0625, 0.125, 0.25, 0.5, and 1 mg/mL) by the CCK‐8 assay. We confirmed that BLF did not induce any cytotoxicity at these concentrations (Figure 1A). Subsequently, we treated PK15 cells with BLF, infected the cells with PRV in the presence of BLF, and incubated the cells in the continued presence of BLF for 24 h, and then measured the viral titers using the plaque assay (Figure 1B). We observed that BLF displayed a strong inhibitory effect on PRV replication, with enhanced antiviral activity as the concentration increases, with significant 11‐fold, 40‐fold, and 65‐fold reductions in PRV titers after treatment with 0.25 mg/mL, 0.5 mg/mL, and 1 mg/mL BLF, respectively (Figure 1C). These results suggested that BLF exerts an antiviral effect against PRV in a dose‐dependent manner.

To ensure that our antiviral assays were performed properly, a reported small‐molecule inhibitor MLN4924 that exhibited a strong inhibitory effect on PRV replication was included in parallel experiments as a positive control [44, 45]. Consistent with the previous findings [44, 45], MLN4924 treatment resulted in a significant 12‐fold reduction in PRV titers (Figure 1D), further validating the feasibility of our antiviral experiments. Then, we determined that BLF has an effective concentration of 107.8 µg/mL inhibiting PRV growth by 50% (EC_50_) (Figure 1E). To determine whether the inhibitory effect of BLF on PRV replication was MOI‐dependent, antiviral experiments were carried out under different MOIs. As expected, the antiviral activity of BLF against PRV was weakened with the increase of MOI, with significant 125‐fold, 63‐fold, and 11‐fold reductions in PRV titers under MOI 0.01, 0.1, and 1, respectively, suggesting that the antiviral efficacy of BLF against PRV is MOI‐dependent (Figure 1F). Taken together, these findings demonstrated that BLF effectively inhibits PRV replication in PK15 cells.

**Figure 2:**
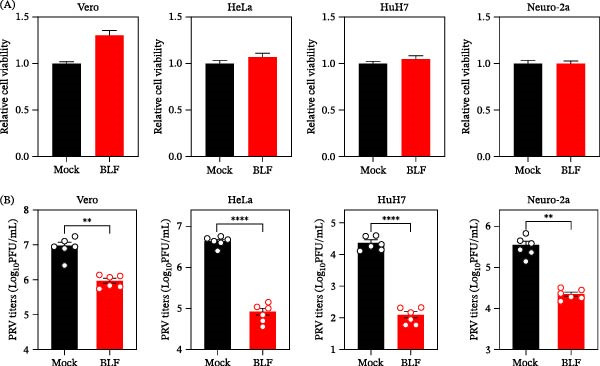
BLF inhibits PRV replication in multiple permissive cells. (A) Vero, HeLa, HuH7, and Neuro‐2a cells were treated with 1 mg/mL BLF for 24 h, then the cell viability was determined by the CCK‐8 assay. (B) Vero, HeLa, HuH7, and Neuro‐2a cells were mock‐treated, or treated with 1 mg/mL BLF for 2 h, infected with PRV at MOI 0.1 (for Vero, HeLa, and HuH7) or 1 (for Neuro‐2a) in the presence of BLF for 1 h, and then incubated in the continued presence of BLF for 24 h. The viral titers in the cells and supernatants were measured by plaque assay. Values represent the means ± the SEM of two independent experiments. Significance was determined by a two‐tailed, unpaired *t*‐test ( ^∗∗^ *p* < 0.01,  ^∗∗∗∗^ *p* < 0.0001).

PRV has a wide host range, with the ability to infect a large variety of cells from humans and other animals. Therefore, in the following experiments, we determined whether BLF inhibited PRV replication in other permissive cells. We chose four other permissive cell lines: African green monkey kidney cells (Vero), human cervical carcinoma cells (HeLa), human hepatoma cells (HuH7), and mouse neuroblastoma cells (Neuro‐2a). We first verified that BLF did not induce any cytotoxicity in these cell lines under the concentration tested (Figure 2A). Then, we determined the antiviral activity of BLF against PRV infection in these cell lines at 24 h postinfection (hpi). In agreement with a significant inhibition of PRV replication observed in PK15 cells (Figure 1), BLF treatment also resulted in a striking repression of PRV replication in these cell lines, with significant 10‐fold, 52‐fold, 182‐fold, and 16‐fold reductions in PRV titers in Vero, HeLa, HuH7, and Neuro‐2a cells, respectively (Figure 2B). Taken together, these findings demonstrated that BLF exerts strong antiviral activity against PRV infection in multiple pig, human, and mouse permissive cells. Cumulatively, these findings revealed BLF as a novel antiviral agent that effectively inhibits PRV infection.

### 3.2. Addition of BLF Before and During Infection Inhibits PRV Replication

**Figure 3:**
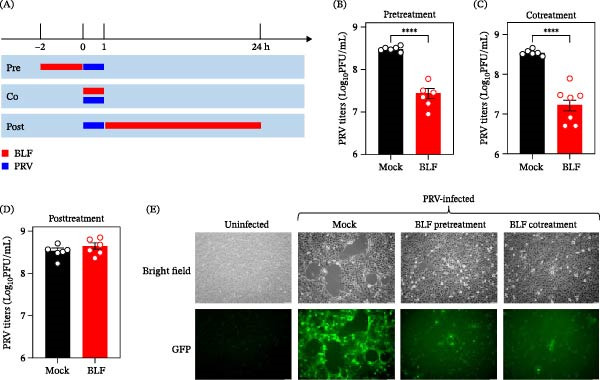
Addition of BLF before and during infection inhibits PRV replication. (A) The schematic diagram of the drug time‐of‐addition assay in PK15 cells. (B) PK15 cells were mock‐treated, or treated with 1 mg/mL BLF at 37°C for 2 h (pretreatment), infected with PRV at MOI 0.1, and then incubated for 24 h. (C) PK15 cells were infected with PRV at MOI 0.1 in the absence or presence of 1 mg/mL BLF at 37°C for 1 h (cotreatment), and then incubated for 24 h. (D) PK15 cells were infected with PRV at MOI 0.1 for 1 h, and then incubated in the absence or presence of 1 mg/mL BLF at 37°C for 24 h (posttreatment). The viral titers in the cells and supernatants were determined by plaque assay. Values represent the means ± the SEM of two independent experiments. Significance was determined by a two‐tailed, unpaired *t*‐test ( ^∗∗∗∗^ *p* < 0.0001). (E) PK15 cells were mock‐treated or treated with 1 mg/mL BLF at 37°C for 2 h (pretreatment), infected with PRV expressing GFP at MOI 0.1 in the absence or presence of 1 mg/mL BLF at 37°C for 1 h (cotreatment), and then incubated for 12 h. The cytopathic effects were visualized by fluorescent microscope, and the bright field and GFP signals are shown.

Having confirmed a strong inhibitory effect of BLF on PRV replication, we next sought to determine the specific stage at which BLF represses PRV. To test this, we carried out the drug time‐of‐addition experiments where BLF was added before, during, or post PRV infection (Figure 3A). Addition of BLF before and during infection was capable of effectively inhibiting PRV replication, with significant 11‐fold and 20‐fold reductions in PRV titers, respectively (Figure 3B,C). In contrast, the addition of BLF after infection did not display any antiviral activity against PRV (Figure 3D). We further validated these results using a PRV recombinant marker virus expressing GFP, observing that the PRV‐induced cytopathic effects and the number of GFP‐positive infected cells were strikingly reduced when BLF was added before and during infection (Figure 3E).

**Figure 4:**
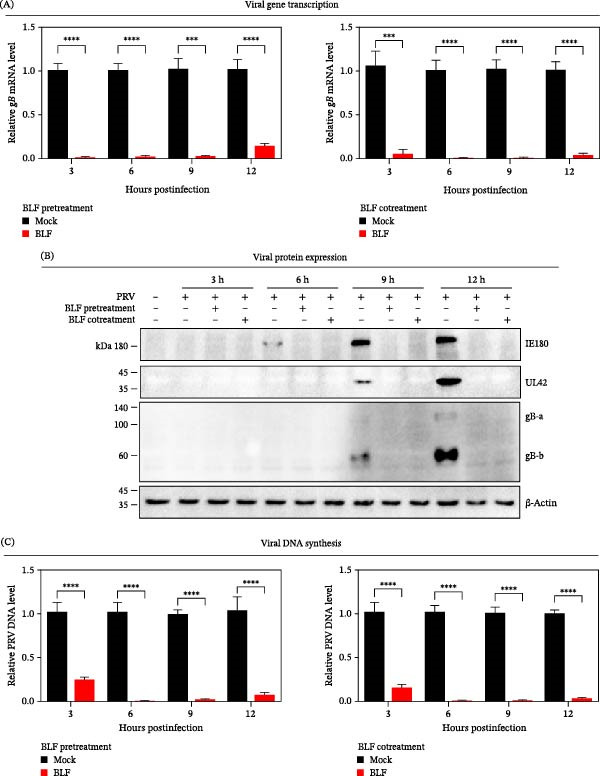
PRV gene expression and DNA synthesis were inhibited in the presence of BLF. PK15 cells were mock‐treated or treated with 1 mg/mL BLF at 37°C for 2 h (pretreatment), infected with PRV at MOI 1 in the absence or presence of 1 mg/mL BLF at 37°C for 1 h (cotreatment), and then incubated for 3, 6, 9, and 12 h. (A) RNA was extracted from the cells, and reversed transcribed, then the mRNA expression levels of PRV *gB* gene were assessed by qPCR. (B) The protein lysates were harvested from the cells, then the expression levels of IE180, UL42, and gB proteins were determined by western blots. β‐Actin was included as a loading control. (C) DNA was extracted from the cells, and the viral gB DNA copies were measured by qPCR. Relative PRV genomic DNA level was expressed as the normalization of viral gB DNA copies in the presence of BLF to those of mock treatment. Values represent the means ± the SEM of two independent experiments. Significance was determined by a two‐tailed, unpaired *t*‐test ( ^∗∗∗^ *p* < 0.001,  ^∗∗∗∗^ *p* < 0.0001).

We next determined the impact of BLF, when added before and during infection, on PRV gene expression and DNA synthesis. As compared to mock‐treated cells, we observed that BLF treatment resulted in a significant reduction in the mRNA expression of the PRV *gB* gene at 3, 6, 9, and 12 hpi (Figure 4A). Consistent with a striking decrease in mRNA levels, BLF treatment also led to a remarkable reduction in the expression of PRV IE180, UL42, and gB proteins (Figure 4B). Furthermore, the synthesis of PRV DNA in the presence of BLF was strikingly impeded at all time points tested (Figure 4C). Taken together, these findings demonstrated that the addition of BLF before and during infection inhibits PRV replication. Cumulatively, these findings suggested that BLF may exert antiviral efficacy against PRV at the virus entry stage.

### 3.3. BLF Represses PRV Attachment to Target Cells

**Figure 5:**
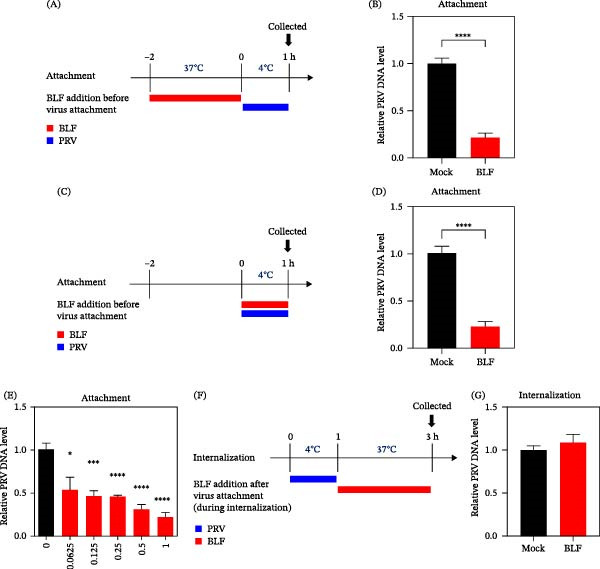
BLF inhibits PRV attachment to target cells. (A) The schematic diagram of virus attachment experiments with BLF addition before virus attachment. (B) PK15 cells were mock‐treated or treated with 1 mg/mL BLF at 37°C for 2 h, then infected with PRV at MOI 1 at 4°C for 1 h. (C) The schematic diagram of virus attachment experiments with BLF addition during virus attachment. (D) PK15 cells were infected with PRV at MOI 1 in the absence or presence of 1 mg/mL BLF at 4°C for 1 h. (E) PK15 cells were infected with PRV at MOI 1 in the presence of 0, 0.0625, 0.125, 0.25, 0.5, or 1 mg/mL BLF at 4°C for 1 h. (F) The schematic diagram of virus internalization experiments with BLF addition after virus attachment (during virus internalization). (G) PK15 cells were infected with PRV at MOI 1 at 4°C for 1 h, then mock‐treated, or treated with 1 mg/mL BLF at 37°C for 2 h. (B, D, E, G) DNA was extracted from the cells, then the viral gB DNA copies were quantified by qPCR. Relative PRV genomic DNA level was expressed as the normalization of viral gB DNA copies in the presence of BLF to those of mock treatment. Values represent the means ± the SEM of two or three independent experiments. Significance was determined by a two‐tailed, unpaired *t*‐test ( ^∗^ *p* < 0.05,  ^∗∗∗^ *p* < 0.001,  ^∗∗∗∗^ *p* < 0.0001).

The results described above demonstrated that BLF effectively inhibits PRV replication when added before and during infection, strongly suggesting that it affects the virus entry stage. Therefore, in the following experiments, we determined whether BLF had an impact on PRV attachment and internalization. We first treated PK15 cells with BLF and then allowed PRV to attach to cells at 4°C (Figure 5A); the virus attached to cells was then measured by qPCR. We observed that BLF pretreatment caused a striking 79% decrease in virus attachment as compared to the mock group (Figure 5B). Then, we infected PK15 cells with PRV in the absence or presence of BLF at 4°C (Figure 5C) and then quantified the virus attached to cells by qPCR. Addition of BLF during infection also resulted in a significant 77% decrease in virus attachment as compared to the mock group (Figure 5D). Moreover, we confirmed that BLF inhibited PRV attachment in a dose‐dependent manner (Figure 5E). Taken together, these results demonstrated that BLF inhibits PRV attachment.

Subsequently, we determined whether BLF affected virus internalization. Following infection of PK15 cells with PRV at 4°C (which allows virus attachment to cells), BLF was added during virus internalization (Figure 5F), and then, qPCR was performed to assess the virus internalized into cells. We found that PRV was internalized into both BLF‐ and mock‐treated cells at very similar levels (Figure 5G), demonstrating that BLF does not have an impact on PRV internalization. Cumulatively, these findings demonstrated that BLF represses PRV attachment to target cells.

### 3.4. BLF Inhibits PRV Attachment by Preventing the Binding of Viral gC Protein to Cell‐Surface HS

**Figure 6:**
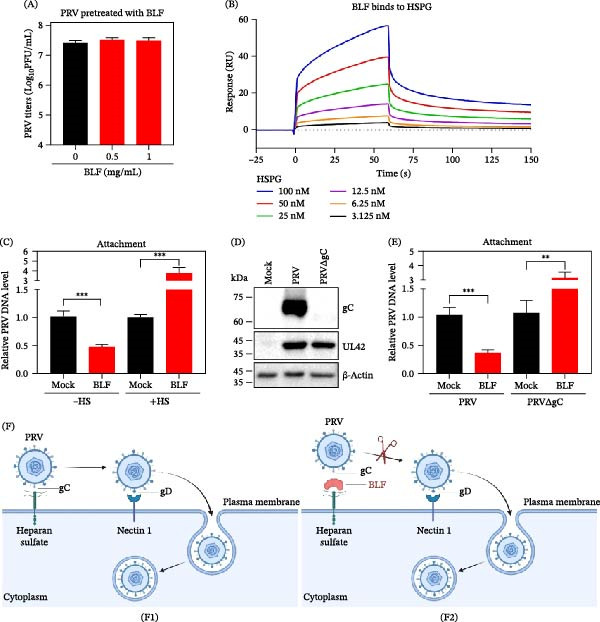
BLF inhibits PRV attachment by preventing the binding of viral gC protein to cell‐surface HS. (A) PRV virions were pretreated with 0, 0.5 or 1 mg/mL BLF at 37°C for 2 h, then the mixtures of PRV and BLF were subjected to plaque assay. (B) SPR analysis of the binding kinetics between BLF and HSPG. (C) Addition of exogenous HS reverses the inhibitory effect of BLF on PRV attachment. BLF was pretreated with or without porcine HS at room temperature for 1 h, then added to PK15 cells and incubated at 37°C for 2 h. The cells were then infected with PRV at 4°C for 1 h. DNA was extracted from the cells, then the viral gB DNA copies were measured by qPCR. Relative PRV genomic DNA level was expressed as the normalization of viral gB DNA copies in the presence of BLF to those of mock treatment. (D) PK15 cells were mock infected, or infected with PRV wild‐type or gC‐deficient (PRVΔgC) viruses for 24 h, then the expression levels of PRV gC and UL42 proteins in PRV or PRVΔgC‐infected cells were detected by western blots. β‐Actin was included as a loading control. (E) BLF fails to inhibit the attachment of PRV gC‐deficient virus. PK15 cells were mock‐treated or treated with 1 mg/mL BLF at 37°C for 2 h, then infected with PRV or PRVΔgC at 4°C for 1 h. DNA was extracted and quantified exactly as described for (C). Values represent the means ± the SEM of two independent experiments. Significance was determined by a two‐tailed, unpaired *t*‐test ( ^∗∗^ *p* < 0.01,  ^∗∗∗^ *p* < 0.001). (F) A proposed working model for BLF inhibition of PRV attachment. (F1) PRV utilizes viral gC protein to bind heparan sulfate (HS) for attachment to target cells, followed by engagement with a specific cell‐surface receptor, such as nectin 1, through viral gD protein, which promotes viral entry into the cytoplasm. (F2) BLF interacts with HS, which prevents the binding of PRV gC to HS and thus inhibits virus attachment.

It has been demonstrated that BLF generally utilizes two mechanisms to defend against viral infection: directly binding to viral particles or interacting with cell‐surface receptors [34–39]. To determine whether BLF was able to bind PRV virions directly, we performed a functional assay to test whether BLF could counteract PRV infectivity. PRV virions were pretreated with BLF, and then viral titers were measured by plaque assay in PK15 cells. We found that BLF‐treated virus produced a similar number of plaques as mock‐treated control virus (Figure 6A), suggesting that there is no direct binding of BLF to PRV virions. These results demonstrated that BLF inhibition of PRV attachment is not achieved by directly binding to viral particles.

We next determined whether BLF inhibited PRV attachment by interacting with specific receptors on the surface of target cells. Because BLF has recently been shown to interact with HSPG to exert antiviral activity [39], and HSPG serves as a primary attachment receptor for PRV [18–22], we next determined whether BLF suppressed PRV attachment by interacting with HSPG. Through SPR analysis, we demonstrated that BLF directly interacted with HSPG (Figure 6B). The equilibrium dissociation constant (*K*_*D*_), association rate constant (Ka), and dissociation rate constant (Kd) for the interaction between BLF and HSPG were 7.41 × 10^−8^ M, 2.60 × 10^5^ M^−1^s^−1^, and 1.93 × 10^−2^ s^−1^, respectively. Having demonstrated the interaction between BLF and HSPG, we next determined whether the addition of exogenous HS could competitively inhibit the effect of BLF on PRV attachment. BLF was pretreated with porcine HS first and then added to PK15 cells, followed by PRV infection. PRV attachment was then measured by qPCR. In agreement with our above findings (Figure 5B), BLF strongly inhibited PRV attachment in the absence of HS (Figure 6C). However, the addition of exogenous HS completely reversed the inhibitory effect of BLF on PRV attachment (Figure 6C). These results suggested that BLF represses PRV attachment by interacting with cell‐surface HS.

Since PRV gC has been identified to be the primary viral glycoprotein that binds cell‐surface HS to mediate virus attachment [18–22] and the virus deficient in the expression of gC enters target cells in a HS‐independent fashion [18, 19], we hypothesized that BLF might block PRV attachment by disrupting the interaction between gC and cell‐surface HS. If this hypothesis were real, then BLF would lose its activity to repress PRV attachment without gC. To test this, the effect of BLF on the attachment of the PRVΔgC virus was determined. Using the western blot assay, we first verified that the PRV gC protein expression has already disappeared in PRVΔgC virus‐infected cells (Figure 6D). Afterwards, we determined the ability of PRV wild‐type and gC‐deficient viruses to attach PK15 cells by qPCR. In agreement with our above findings (Figure 5B), BLF exhibited a significant inhibitory effect on the attachment of the PRV wild‐type virus (Figure 6E). However, BLF completely lost its ability to inhibit the attachment of PRVΔgC virus (Figure 6E), suggesting that gC is required for BLF inhibition of PRV attachment. Cumulatively, these findings demonstrated that BLF interacts with cell‐surface HS and thus inhibits PRV attachment by interfering with the binding of viral gC to HS (Figure 6F).

## 4. Discussion

PRV infection not only results in severe clinical diseases in pigs but also gradually becomes a potential threat to veterinary public health. Therefore, to prepare for possible future prevalence, the development of effective drugs against PRV infection and spread is needed. In the work described here, BLF was demonstrated as a novel and potent antiviral agent against PRV infection. BLF not only effectively inhibited PRV replication in PK15 cells but also significantly repressed PRV replication in multiple other permissive cells, including Vero, HeLa, HuH7, and Neuro‐2a. Systematic investigation on the effect of BLF on PRV inhibition demonstrated that BLF blocks PRV attachment to target cells. SPR analysis revealed that BLF directly interacted with HSPG, and addition of exogenous HS completely abolished BLF inhibition of PRV attachment, suggesting that BLF represses PRV attachment by interacting with cell‐surface HS. However, BLF failed to inhibit the attachment of the PRVΔgC virus to target cells. Cumulatively, these findings revealed that BLF inhibits PRV attachment by interacting with cell‐surface HS and preventing the binding of PRV gC to HS.

BLF is widely recognized as an iron‐binding glycoprotein in the transferrin family. It exhibits broad‐spectrum biological activities, including iron delivery, antioxidant, anti‐inflammatory, antitumor, antimicrobial, antiviral, immunomodulatory, and prebiotic functions [29]. These excellent features make it have significant application value in the fields of biomedicine and biotechnology. The first important and successful application of BLF in a commercial product was its key role as an integral ingredient in infant formula, which helps narrow the nutritional gap with human breast milk by supporting immune function and healthy gut development in infants [30]. Subsequently, BLF was applied for dietary supplements and functional food formulations. For example, BLF was used as a supplement for probiotic foods to improve beneficial intestinal flora and for functional foods to promote iron absorption. Furthermore, BLF can be used as a nutraceutical to inhibit the host inflammatory response and enhance immune function [30]. Notably, a greatly growing demand for BLF production has recently been observed for infant formulas, dietary supplements, and functional food formulations [29]. In addition, because BLF can delay lipid oxidation, it has potential as a preservative in food storage and cosmetics [46, 47]. Taken together, BLF is a versatile nutraceutical milk protein, a food‐derived substance with biomedical benefits.

It is noteworthy that BLF has been demonstrated to display potent antiviral activities against a variety of viruses, such as HSV1, HSV2, SARS‐CoV, SARS‐CoV‐2, HIV, and HBV [31–33]. In agreement with these exciting previous findings, here, we also confirmed that BLF exhibits potent antiviral efficacy against PRV infection in multiple permissive cells, further increasing its antiviral range. During the coronavirus disease 2019 (COVID‐19) pandemic, the researchers across the world made tremendous efforts to find novel therapeutics for SARS‐CoV‐2, and BLF gained great attention due to its potential effects on health. Several research groups independently demonstrated that BLF could effectively inhibit the infections of SARS‐CoV‐2 and its variants [39, 48–52]. Moreover, it has recently been demonstrated that BLF displayed strong antiviral activity against several animal viruses, including porcine epidemic diarrhea virus (PEDV), senecavirus A (SVA), foot and mouth disease virus (FMDV), and bovine viral diarrhea virus (BVDV) [40, 53, 54]. Taken together, these findings demonstrated that BLF is a promising prophylactic and therapeutic agent against COVID‐19 and other virus‐associated diseases [55–57].

Generally, BLF employs two mechanisms to defend against viral infection [34–40]. The first mechanism is accomplished by interacting with specific cell‐surface receptors, which impairs virus attachment [37–40]. For example, BLF blocked dengue virus infection by interacting with cell‐surface HS, dendritic cell‐specific intercellular adhesion molecule 3‐grabbing nonintegrin (DC‐SIGN), and low‐density lipoprotein receptors (LDLRs) [38]. Moreover, BLF exhibited potent antiviral activity against PEDV by interacting with HSPG and interfering with the binding of viral spike (S) protein to HSPG [40]. Consistent with these findings, we demonstrated that BLF inhibits PRV infection by interacting with cell‐surface HS and interfering with the binding of viral gC to HS. The property of BLF binding of cell‐surface receptors makes it a promising and broad‐spectrum antiviral agent against viruses. The second mechanism is achieved by directly binding to viral particles and the inhibition of virus attachment to target cells [34–36]. For example, BLF utilized its C‐lobe domain to interact with influenza A virus hemagglutinin, thus interfering with its fusogenic function and preventing infection by different H1 and H3 viral subtypes [34, 35]. Furthermore, BLF bound the receptor‐binding domain of SARS‐CoV‐2 S protein through its N‐terminus, which prevented the binding of S protein to cell‐surface receptors angiotensin‐converting enzyme‐2 (ACE2) and HSPG [36]. Interestingly, aside from these two mechanisms, BLF could inhibit the replication of SARS‐CoV and SARS‐CoV‐2 by inhibiting RNA‐dependent RNA polymerase activity [50]. These results demonstrated that, in addition to its universal antiviral action by binding cell‐surface receptors, BLF also displays a virus‐specific antiviral action by interacting with viral proteins to block virus attachment or interfering with the functions of certain viral proteins. Taken together, these findings suggested that BLF may utilize multiple strategies to combat virus infection, which is largely dependent on the species of the virus.

A surprising phenomenon that we found in this study was that BLF appeared to induce enhancement of PRV attachment when BLF was first incubated with exogenous HS (Figure 6C) or the PRV *gC* gene was deleted from the virus (Figure 6E). It is well‐known that HS itself displays an inhibitory effect on PRV attachment, and we demonstrate here that BLF directly binds HS. Therefore, preincubation of HS with BLF counteracts the inhibitory effect of HS on PRV attachment, which results in an increase in PRV attachment when compared to the only HS‐treated cells. The specific mechanism by which BLF elevates the attachment of PRVΔgC virus remains unclear, but we hypothesize that when the primary PRV gC binding site is disabled, BLF may activate an alternative route to facilitate virus entry. It will be interesting to define the specific alternative route by which BLF promotes the attachment of the PRVΔgC virus.

## 5. Conclusions

In summary, the findings presented here demonstrate BLF as a novel antiviral agent that effectively represses PRV infection. BLF exerts an inhibitory effect on PRV attachment by interacting with cell‐surface HS and interfering with the binding of viral gC to HS. This work demonstrates that BLF, as a multifunctional biomolecule and a well‐known nutraceutical milk protein, serves as an effective and promising antiviral drug candidate against PRV infection.

## Author Contributions

**Yiping Wang**: conceptualization, investigation, methodology, formal analysis, data curation, writing – original draft, writing – review and editing, supervision, project administration, funding acquisition. **Binbin Zhu**: investigation, methodology, validation, data curation, visualization. **Fei Zhao**: investigation, data curation. **Zhiyuan Zheng and Senhong Zhao**: investigation. **Yi Zheng, Xiaobo Huang, Qin Zhao, Senyan Du, Yiping Wen, and Rui Wu**: resources. **Tongqing An and Sanjie Cao**: formal analysis, writing – review and editing, supervision, project administration, funding acquisition.

## Funding

This work was supported by the State Key Laboratory of Animal Disease Control and Prevention Foundation (Grant SKLADCPKFKT202404), the China Postdoctoral Science Foundation (Grant 2025T180830), the Sichuan Tianfu Emei Program (Grant 2610), and the Chengdu Rongpiao Program (Grant 1163).

## Ethics Statement

This study does not involve any experiments on animals or humans; thus, ethical approval is not required.

## Conflicts of Interest

The authors declare no conflicts of interest.

## Data Availability

The data that support the findings of this study are available from the corresponding author upon reasonable request.

## References

[1] Pomeranz L. E. , Reynolds A. E. , and Hengartner C. J. , Molecular Biology of Pseudorabies Virus: Impact on Neurovirology and Veterinary Medicine, Microbiology and Molecular Biology Reviews. (2005) 69, no. 3, 462–500, 10.1128/MMBR.69.3.462-500.2005. 16148307 PMC1197806

[2] Bo Z. and Li X. , A Review of Pseudorabies Virus Variants: Genomics, Vaccination, Transmission, and Zoonotic Potential, Viruses. (2022) 14, no. 5, 10.3390/v14051003, 1003. 35632745 PMC9144770

[3] Marcaccini A. , Peña M. L. , Quiroga M. I. , Bermúdez R. , Nieto J. M. , and Alemañ N. , Pseudorabies Virus Infection in Mink: A Host-Specific Pathogenesis, Veterinary Immunology and Immunopathology. (2008) 124, no. 3-4, 264–273, 10.1016/j.vetimm.2008.03.013. 18490062

[4] Avak S. , Bienzle U. , Feldmeier H. , Hampl H. , and Habermehl K.-O. , Pseudorabies in Man, The Lancet. (1987) 329, no. 8531, 501–502, 10.1016/S0140-6736(87)92105-2. 2881053

[5] Skinner G. R. B. , Ahmad A. , and Davies J. A. , The Infrequency of Transmission of Herpesviruses Between Humans and Animals; Postulation of an Unrecognised Protective Host Mechanism, Comparative Immunology, Microbiology and Infectious Diseases. (2001) 24, no. 4, 255–269, 10.1016/S0147-9571(01)00014-5. 11561960

[6] Ai J.-W. , Weng S.-S. , and Cheng Q. , et al.Human Endophthalmitis Caused by Pseudorabies Virus Infection, China, 2017, Emerging Infectious Diseases. (2018) 24, no. 6, 1087–1090, 10.3201/eid2406.171612. 29774834 PMC6004832

[7] Yang X. , Guan H. , and Li C. , et al.Characteristics of Human Encephalitis Caused by Pseudorabies Virus: A Case Series Study, International Journal of Infectious Diseases. (2019) 87, 92–99, 10.1016/j.ijid.2019.08.007. 31408708

[8] Wang Y. , Nian H. , Li Z. , Wang W. , Wang X. , and Cui Y. , Human Encephalitis Complicated With Bilateral Acute Retinal Necrosis Associated With Pseudorabies Virus Infection: A Case Report, International Journal of Infectious Diseases. (2019) 89, 51–54, 10.1016/j.ijid.2019.09.019. 31562933

[9] Yang H. N. , Han H. , Wang H. , Cui Y. , Liu H. , and Ding S. F. , A Case of Human Viral Encephalitis Caused by Pseudorabies Virus Infection in China, Frontiers in Neurology. (2019) 10, 10.3389/fneur.2019.00534, 534. 31214104 PMC6558170

[10] Fan S. , Yuan H. , and Liu L. , et al.Pseudorabies Virus Encephalitis in Humans: A Case Series Study, Journal of NeuroVirology. (2020) 26, no. 4, 556–564, 10.1007/s13365-020-00855-y. 32572833

[11] Wang D. , Tao X. , and Fei M. , et al.Human Encephalitis Caused by Pseudorabies Virus Infection: A Case Report, Journal of NeuroVirology. (2020) 26, no. 3, 442–448, 10.1007/s13365-019-00822-2. 31898060 PMC7223082

[12] Li X. D. , Fu S. H. , and Chen L. Y. , et al.Detection of Pseudorabies Virus Antibodies in Human Encephalitis Cases, Biomedical and Environmental Sciences. (2020) 33, no. 6, 444–447. 32641207 10.3967/bes2020.059

[13] Liu Q. , Wang X. , and Xie C. , et al.A Novel Human Acute Encephalitis Caused by Pseudorabies Virus Variant Strain, Clinical Infectious Diseases. (2021) 73, no. 11, e3690–e3700, 10.1093/cid/ciaa987. 32667972

[14] Wong G. , Lu J. , Zhang W. , and Gao G. F. , Pseudorabies Virus: A Neglected Zoonotic Pathogen in Humans?, Emerging Microbes & Infections. (2019) 8, no. 1, 150–154, 10.1080/22221751.2018.1563459. 30866769 PMC6455137

[15] Guo Z. , Chen X.-X. , and Zhang G. , Human PRV Infection in China: An Alarm to Accelerate Eradication of PRV in Domestic Pigs, Virologica Sinica. (2021) 36, no. 4, 823–828, 10.1007/s12250-021-00347-1. 33538947 PMC8379330

[16] Mettenleiter T. C. , Aujeszky’s Disease (pseudorabies) Virus: The Virus and Molecular Pathogenesis—State of the Art, June 1999, Veterinary Research. (2000) 31, no. 1, 99–115, 10.1051/vetres:2000059. 10726640

[17] Klupp B. G. , Hengartner C. J. , Mettenleiter T. C. , and Enquist L. W. , Complete, Annotated Sequence of the Pseudorabies Virus Genome, Journal of Virology. (2004) 78, no. 4, 2166–2166, 10.1128/JVI.78.4.2166.2004. PMC303424 14671123

[18] Mettenleiter T. C. , Zsak L. , Zuckermann F. , Sugg N. , Kern H. , and Ben-Porat T. , Interaction of Glycoprotein GIII With a Cellular Heparinlike Substance Mediates Adsorption of Pseudorabies Virus, Journal of Virology. (1990) 64, no. 1, 278–286, 10.1128/jvi.64.1.278-286.1990. 2152816 PMC249100

[19] Karger A. , Saalmüller A. , Tufaro F. , Banfield B. W. , and Mettenleiter T. C. , Cell Surface Proteoglycans Are Not Essential for Infection by Pseudorabies Virus, Journal of Virology. (1995) 69, no. 6, 3482–3489, 10.1128/jvi.69.6.3482-3489.1995. 7745695 PMC189061

[20] Zsak L. , Sugg N. , Ben-Porat T. , Robbins A. K. , Whealy M. E. , and Enquist L. W. , The gIII Glycoprotein of Pseudorabies Virus Is Involved in Two Distinct Steps of Virus Attachment, Journal of Virology. (1991) 65, no. 8, 4317–4324, 10.1128/jvi.65.8.4317-4324.1991. 1649332 PMC248870

[21] Karger A. and Mettenleiter T. C. , Identification of Cell Surface Molecules That Interact With Pseudorabies Virus, Journal of Virology. (1996) 70, no. 4, 2138–2145, 10.1128/jvi.70.4.2138-2145.1996. 8642635 PMC190051

[22] Trybala E. , Bergström T. , Spillmann D. , Svennerholm B. , Flynn S. J. , and Ryan P. , Interaction Between Pseudorabies Virus and Heparin/Heparan Sulfate: Pseudorabies Virus Mutants Differ in Their Interaction With Heparin/Heparan Sulfate When Altered for Specific Glycoprotein C Heparin-Binding Domain, Journal of Biological Chemistry. (1998) 273, no. 9, 5047–5052, 10.1074/jbc.273.9.5047. 9478954

[23] Milne R. S. B. , Connolly S. A. , Krummenacher C. , Eisenberg R. J. , and Cohen G. H. , Porcine HveC, a Member of the Highly Conserved HveC/Nectin 1 Family, Is a Functional Alphaherpesvirus Receptor, Virology. (2001) 281, no. 2, 315–328, 10.1006/viro.2000.0798. 11277703

[24] Li A. , Lu G. , and Qi J. , et al.Structural Basis of Nectin-1 Recognition by Pseudorabies Virus Glycoprotein D, PLoS Pathogens. (2017) 13, no. 5, 10.1371/journal.ppat.1006314. PMC5453625 28542478

[25] Ye N. , Feng W. , Fu T. , Tang D. , Zeng Z. , and Wang B. , Membrane Fusion, Potential Threats, and Natural Antiviral Drugs of Pseudorabies Virus, Veterinary Research. (2023) 54, no. 1, 10.1186/s13567-023-01171-z. PMC10152797 37131259

[26] Backovic M. , DuBois R. M. , and Cockburn J. J. , et al.Structure of a Core Fragment of Glycoprotein H From Pseudorabies Virus in Complex With Antibody, Proceedings of the National Academy of Sciences. (2010) 107, no. 52, 22635–22640, 10.1073/pnas.1011507107. PMC3012528 21149698

[27] Li X. , Zheng J. , and Lv X. , et al.Vimentin as a Universal Receptor for Pseudorabies Virus Infection in Pig and Human Cells, International Journal of Biological Macromolecules. (2024) 283, 10.1016/j.ijbiomac.2024.137638, 137638. 39549807

[28] Azab W. and Osterrieder K. , Osterrieder K. , Initial Contact: The First Steps in Herpesvirus Entry, Advances in Anatomy Embryology and Cell Biology, 2017, 223, Springer, Cham, 1–27. 28528437 10.1007/978-3-319-53168-7_1

[29] Dyrda-Terniuk T. and Pomastowski P. , The Multifaceted Roles of Bovine Lactoferrin: Molecular Structure, Isolation Methods, Analytical Characteristics, and Biological Properties, Journal of Agricultural and Food Chemistry. (2023) 71, no. 51, 20500–20531, 10.1021/acs.jafc.3c06887. 38091520 PMC10755757

[30] Superti F. , Lactoferrin From Bovine Milk: A Protective Companion for Life, Nutrients. (2020) 12, no. 9, 10.3390/nu12092562, 2562. 32847014 PMC7551115

[31] Eker F. , Duman H. , Ertürk M. , and Karav S. , The Potential of Lactoferrin as Antiviral and Immune-Modulating Agent in Viral Infectious Diseases, Frontiers in Immunology. (2024) 15, 10.3389/fimmu.2024.1402135, 1402135. 39620218 PMC11604709

[32] Shafqat A. , Li M. , and Zakirullah , A Comprehensive Review of Research Advances in the Study of Lactoferrin to Treat Viral Infections, Life Sciences. (2025) 361, 10.1016/j.lfs.2024.123340, 123340. 39730037

[33] Kell D. B. , Heyden E. L. , and Pretorius E. , The Biology of Lactoferrin, an Iron-Binding Protein That Can Help Defend Against Viruses and Bacteria, Frontiers in Immunology. (2020) 11, 10.3389/fimmu.2020.01221, 1221. 32574271 PMC7271924

[34] Ammendolia M. G. , Agamennone M. , and Pietrantoni A. , et al.Bovine Lactoferrin-Derived Peptides as Novel Broad-Spectrum Inhibitors of Influenza Virus, Pathogens and Global Health. (2013) 106, no. 1, 12–19, 10.1179/2047773212Y.0000000004. PMC4001507 22595270

[35] Superti F. , Agamennone M. , Pietrantoni A. , and Ammendolia M. G. , Bovine Lactoferrin Prevents Influenza A Virus Infection by Interfering With the Fusogenic Function of Viral Hemagglutinin, Viruses. (2019) 11, no. 1, 10.3390/v11010051, 51. 30641890 PMC6357187

[36] Babulic P. , Cehlar O. , Ondrovičová G. , Moskalets T. , Skrabana R. , and Leksa V. , Lactoferrin Binds through Its N-Terminus to the Receptor-Binding Domain of the SARS-CoV-2 Spike Protein, Pharmaceuticals. (2024) 17, no. 8, 10.3390/ph17081021, 1021. 39204126 PMC11357225

[37] Lang J. , Yang N. , and Deng J. , et al.Inhibition of SARS Pseudovirus Cell Entry by Lactoferrin Binding to Heparan Sulfate Proteoglycans, PLoS ONE. (2011) 6, no. 8, 10.1371/journal.pone.0023710. PMC3161750 21887302

[38] Chen J.-M. , Fan Y.-C. , Lin J.-W. , Chen Y.-Y. , Hsu W.-L. , and Chiou S.-S. , Bovine Lactoferrin Inhibits Dengue Virus Infectivity by Interacting With Heparan Sulfate, Low-Density Lipoprotein Receptor, and DC-SIGN, International Journal of Molecular Sciences. (2017) 18, no. 9, 10.3390/ijms18091957, 1957. 28895925 PMC5618606

[39] Hu Y. , Meng X. , Zhang F. , Xiang Y. , and Wang J. , The *In Vitro* Antiviral Activity of Lactoferrin Against Common Human Coronaviruses and SARS-CoV-2 Is Mediated by Targeting the Heparan Sulfate Co-Receptor, Emerging Microbes & Infections. (2021) 10, no. 1, 317–330, 10.1080/22221751.2021.1888660. 33560940 PMC7919907

[40] Liu P. , Zuo J. , Lu H. , Zhang B. , and Wu C. , Lactoferrin Exhibits PEDV Antiviral Activity by Interfering With Spike-Heparan Sulfate Proteoglycans Binding and Activating Mucosal Immune Response, Veterinary Research. (2025) 56, no. 1, 10.1186/s13567-025-01456-5, 25. 39891300 PMC11786531

[41] Wang Y. , Zhu B. , and Huang X. , et al.Aminoquinoline Surfen Inhibits Pseudorabies Virus Attachment by Preventing the Binding of Glycoprotein C to Heparan Sulfate, Microbiology Spectrum. (2026) 14, no. 4, 10.1128/spectrum.03223-25. PMC13055252 41784488

[42] Schmittgen T. D. and Livak K. J. , Analyzing Real-Time PCR Data by the Comparative CT Method, Nature Protocols. (2008) 3, no. 6, 1101–1108, 10.1038/nprot.2008.73. 18546601

[43] Du W. , Wang Y. , and Huang L. , et al.Characterization of Monoclonal Antibodies That Recognize the Amino- and Carboxy-Terminal Epitopes of the Pseudorabies Virus UL42 Protein, Applied Microbiology and Biotechnology. (2016) 100, no. 1, 181–192, 10.1007/s00253-015-6957-7. 26377421

[44] Lee B. H. , Tebaldi G. , Pritchard S. M. , Nicola A. V. , and Jones C. J. , Host Cell Neddylation Facilitates Alphaherpesvirus Entry in a Virus-Specific and Cell-Dependent Manner, Microbiology Spectrum. (2022) 10, no. 5, 10.1128/spectrum.03114-22. PMC9603186 36173301

[45] Zheng Z. , Zhao F. , and Zhao S. , et al.The Ubiquitin Conjugating Enzyme E2 F (UBE2F)-RING-Box Protein 2 (RBX2)-Mediated Neddylation of Cullin5 Facilitates Pseudorabies Virus Replication, International Journal of Biological Macromolecules. (2026) 339, 10.1016/j.ijbiomac.2025.150018, 150018. 41478481

[46] Medina I. , Tombo I. , Satué-Gracia M. T. , German J. B. , and Frankel E. N. , Effects of Natural Phenolic Compounds on the Antioxidant Activity of Lactoferrin in Liposomes and Oil-in-Water Emulsions, Journal of Agricultural and Food Chemistry. (2002) 50, no. 8, 2392–2399, 10.1021/jf011126y. 11929302

[47] Oda H. , Miyakawa M. , and Mizuki M. , et al.Effects of Lactoferrin on Subjective Skin Conditions in Winter: A Preliminary, Randomized, Double-Blinded, Placebo-Controlled Trial, Clinical, Cosmetic and Investigational Dermatology. (2019) 12, 875–880, 10.2147/CCID.S228153. 31819585 PMC6896904

[48] Campione E. , Lanna C. , and Cosio T. , et al.Lactoferrin Against SARS-CoV-2: *In Vitro* and *In Silico* Evidences, Frontiers in Pharmacology. (2021) 12, 10.3389/fphar.2021.666600, 666600. 34220505 PMC8242182

[49] Wotring J. W. , Fursmidt R. , Ward L. , and Sexton J. Z. , Evaluating the In Vitro Efficacy of Bovine Lactoferrin Products Against SARS-CoV-2 Variants of Concern, Journal of Dairy Science. (2022) 105, no. 4, 2791–2802, 10.3168/jds.2021-21247. 35221061 PMC8872794

[50] He S.-T. , Qin H. , and Guan L. , Bovine Lactoferrin Inhibits SARS-CoV-2 and SARS-CoV-1 by Targeting the RdRp Complex and Alleviates Viral Infection in the Hamster Model, Journal of Medical Virology. (2023) 95, no. 1, 10.1002/jmv.28281. PMC9878033 36329614

[51] Alves N. S. , Azevedo A. S. , and Dias B. M. , et al.Inhibition of SARS-CoV-2 Infection in Vero Cells by Bovine Lactoferrin Under Different Iron-Saturation States, Pharmaceuticals. (2023) 16, no. 10, 10.3390/ph16101352, 1352. 37895823 PMC10609673

[52] Kobayashi-Sakamoto M. , Maeda T. , and Yusa J. , et al.Bovine Lactoferrin Suppresses the Cathepsin-Dependent Pathway of SARS-CoV-2 Entry In Vitro, International Dairy Journal. (2024) 148, 10.1016/j.idairyj.2023.105805, 105805.

[53] Cui H. , Li S. , Yan X. , Wang Z. , Leng T. , Li M. , and Li S. , In Vitro Efficacy of Bovine Lactoferrin Against Bovine Viral Diarrhea Virus, Archives of Microbiology. (2025) 207, no. 6, 10.1007/s00203-025-04328-w. 40261405

[54] Zhao W. , Zhang S. , and Sui L. , et al.Inhibitory Effects of Bovine Lactoferricin-Lactoferrampin on Senecavirus A and Foot-and-Mouth Disease Virus With Recombinant Lactobacillus Oral Treatment in Mice, Veterinary Sciences. (2025) 12, no. 3, 10.3390/vetsci12030199, 199. 40266921 PMC11945493

[55] Einerhand A. W. C. , van Loo-Bouwman C. A. , and Weiss G. A. , et al.Can Lactoferrin, a Natural Mammalian Milk Protein, Assist in the Battle Against COVID-19?, Nutrients. (2022) 14, no. 24, 10.3390/nu14245274, 5274. 36558432 PMC9782828

[56] Ward J. L. , Torres-Gonzalez M. , and Ammons M. C. B. , The Influence of Viral Infections on Iron Homeostasis and the Potential for Lactoferrin as a Therapeutic in the Age of the SARS-CoV-2 Pandemic, Nutrients. (2022) 14, no. 15, 10.3390/nu14153090, 3090. 35956266 PMC9370565

[57] Kobayashi-Sakamoto M. , Maeda T. , Yusa J. , Tani H. , Kato Y. , and Hirose K. , Lactoferrin as a Possible Preventive and Therapeutic Agent Against SARS-CoV-2 Infection, Journal of Disaster Research. (2023) 18, no. 1, 27–33, 10.20965/jdr.2023.p0027.

